# Larger Acute Phase Reactions Are Associated with Immunogenicity of an Adjuvanted Recombinant Receptor Binding Domain Protein Vaccine Against SARS-CoV-2 in Rhesus Monkeys

**DOI:** 10.3390/vaccines14060523

**Published:** 2026-06-11

**Authors:** Christopher L. Coe, Gabriele R. Lubach, Francesca Nimityongskul, Kimberly Luke, Eva G. Rakasz, David M. Rancour, Fritz M. Schomburg

**Affiliations:** 1 Harlow Center for Biological Psychology, University of Wisconsin-Madison, Madison, WI 53715, USA; grlubach@wisc.edu; 2 Biostatistics and Medical Informatics, Madison, WI 53792, USA; norante@wisc.edu; 3 Research and Development, Intuitive Biosciences, Madison, WI 53717, USA; kluke@intuitivebio.com; 4 UW AIDS Vaccine Research Laboratory, Madison, WI 53711, USA; egrakasz@wisc.edu; 5 Lytic Solutions, LLC, Madison, WI 53713, USA; david.rancour@lyticsolutions.com (D.M.R.);

**Keywords:** SARS-CoV-2, vaccine, ACE2 binding inhibition, acute phase reactants, iron, neutrophil lymphocyte ratio, rhesus monkey

## Abstract

Background: Although prolonged inflammatory symptoms are an infrequent and problematic adverse effect of vaccination that can occur in some people, the transient activation of acute phase reactants (APRs) is expected with adjuvanted vaccines and helps to potentiate immune responses. Methods: This experiment examined the association between vaccine reactogenicity and immunogenicity in monkeys immunized with an adjuvanted recombinant protein including a receptor binding domain–human IgG1-Fc fusion protein (RBD-Fc) sequenced from the ancestral Wuhan strain of SARS-CoV-2. The acute inflammatory reaction to immunization was assessed by determining the decline in serum iron levels at 24 h and the increase in the neutrophil-to-lymphocyte ratio (NLR) as the adherent neutrophil pool trafficked into circulation. Results: Robust primary and secondary antibody responses were elicited. Larger decreases in serum iron and higher NLRs were associated with a stronger inhibition of RBD binding with angiotensin-converting enzyme (ACE2) when five early viral variants of SARS-CoV-2 were tested, including Wuhan, Alpha, Beta, Gamma and Delta. Inhibition of ACE2-RBD binding was less evident when the Omicron variant was tested. Individual variation in the APR was also predictive of the persistence of cell-mediated immunity based on the number of interferon-expressing mononuclear cells activated by viral antigen in ELISpot assays. Conclusions: Rapid antibody responses to primary immunization and large secondary responses to booster immunizations were elicited by this adjuvanted recombinant RBD-Fc vaccine, and our analysis affirmed the view that a transient APR can enhance antibody binding with antigen proteins.

## 1. Introduction

The occurrence of adverse symptoms in some people after vaccination as well as misinformation about the potential for mRNA vaccines to become incorporated into the somatic genome have contributed to vaccine hesitancy, especially in the United States [1,2]. While health concerns about prolonged inflammatory reactions are valid, especially if directed against cardiovascular tissue, this pathophysiology is limited primarily to a subset of individuals with certain risk factors and inherent propensities [3,4,5,6,7]. Erroneous information has also been disseminated about the safety and functions of the widely used adjuvants and additives that have been included in vaccine formulations for nearly 100 years to facilitate antigen recognition and potentiate immune responses [8,9,10]. The following experiment examined the association between the initial reactogenicity of the host to an adjuvanted recombinant protein vaccine and the immunogenicity elicited against SARS-CoV-2.

The acute phase reaction (APR) is a critical component of the body’s early warning systems, which signals the immune system about the presence of infectious pathogens, facilitates recovery after tissue damage, and helps to identify and control neoplasia. The APR mobilizes innate immune responses and can initiate and augment adaptive immune responses [11]. Some proteins released by the liver, including C-reactive protein (CRP), facilitate antigen recognition and can catalyze antigen–antibody binding. The APR includes a diverse array of other positive and negative responders. For example, there has been increased clinical interest in the sensitivity of the neutrophil-to-lymphocyte ratio (NLR) as a predictor of disease severity and health outcomes [12]. The neutrophilia and elevated NLR reflect the shift of the marginated pool of adherent neutrophils into the circulating pool as they traffic from the vasculature and lungs to the site of infection or injury. The NLR has also been found to be of value for predicting morbidity and the likelihood of mortality in patients with severe COVID [13,14,15]. We employed the transient increase in the NLR after immunization as a positive bioindicator of the reaction to the viral antigen and adjuvant present in our vaccine.

In addition, we focused on acute changes in iron metabolism and regulation as a negative bioindicator of the APR. It has been known for decades that serum iron levels decline quickly after infection and trauma, which reflects the intracellular sequestration of iron as ferritin in monocytes and tissue, including the liver and muscle [16,17]. One adaptive benefit is to limit the bioavailability of iron for bacterial pathogens. In addition, lower levels of iron in circulation may moderate oxidative reactions and lessen damage to healthy tissue from the superoxide and hydrogen peroxide released by activated monocytes and neutrophils [18]. The proinflammatory cytokine, interleukin-6, is a primary mediator of this hypoferremia, helping to induce iron retention in the reticulo-endothelial system and promoting the hepatic release of hepcidin, which limits the absorption and transfer of iron from food in the small intestines [19]. Acute changes in iron-related indices and the NLR were found to be highly correlated in surgical patients undergoing cholecystectomies, and serum iron proved to be more sensitive than serum ferritin for delineating the invasiveness of an open surgical approach as compared to laparoscopic surgery [20]. Further, when the temporal trajectory of the decline in serum iron was tracked after administration of lipopolysaccharides to human participants, iron levels were significantly decreased by 6 h and remained low at 24 h [21]. This time course is ideal for employing serum iron to assess individual variation in the APR after vaccination.

Experiments with relevant animal models may help to inform and lessen some of the prevailing concerns about the vaccines against SARS-CoV-2, especially with respect to the transient inflammatory reactions that occur when purified and recombinant viral proteins are presented in adjuvanted preparations to enhance their immunostimulatory potential [22]. We tested our vaccine in monkeys because they are susceptible to SARS-CoV-2, and it is known that they can mount protective immune responses when vaccinated [23,24,25,26]. Our vaccine included recombinant SARS-CoV-2 RBD–human IgG1-Fc containing protein sequences from the ancestral Wuhan variant. We determined whether individual variation in the APR after immunization was associated with how well the elicited antibody recognized RBD from multiple viral variants, including the ancestral Wuhan strain as well as the Omicron variant. The recombinant protein was administered in either an alum hydroxide suspension (Al(OH)_3_), one of the many aluminum salts used in vaccines for nearly a century [27], or in a suspension with AS03, an oil-in-water emulsion used in some vaccines including the influenza vaccine since the 1990s [28]. Al(OH)_3_ is believed to act more locally, forming a colloidal suspension depot at the injection site, and polarizes for a Th2-dominated immune response. In contrast, the immunogenic actions of AS03 are thought to involve more cytokine and chemokine pathways that facilitate the recruitment of antigen-presenting cells, and it results in a more mixed Th1/Th2 immune response [29]. A comparison of the effectiveness of five different adjuvants administered to monkeys in a study of SARS-CoV-2 RBD presented on a two-component nanoparticle indicated that AS03 elicited more virus neutralization activity, a stronger CD4+ T-cell response, and the most crossover protection against SARS-CoV-2 variants [30]. In our study, biological activity of the antibody against SARS-CoV-2 was assessed with an ACE2-SARS-CoV-2 RBD binding inhibition assay. In addition, we examined cell-mediated immunity by quantifying antigen-reactive, interferon-positive peripheral blood mononuclear cells (PBMCs) with an Enzyme-Linked ImmunoSpot protocol (ELISpot). We evaluated if individual variation in the magnitude of the APR at 24 h after immunization would be associated with antibody inhibition of ACE2-RBD binding and the persistence of T-cell memory. If confirmed, the findings would concur with the view that there is often a concordance between vaccine reactogenicity and immunogenicity [31,32,33,34,35].

## 2. Methods

### 2.1. Subjects

Twelve adult female rhesus monkeys (*Macaca mulatta*, Indian ancestry), 5–11 years of age (5.2–10.1 kg), were immunized against SARS-CoV-2. The monkeys were descendants of a large, genetically diverse founder population imported from India over 50 years ago [36]. The current breeding colony has over 450 monkeys and is housed in an indoor facility with standardized husbandry conditions. Blood from 4 additional female monkeys provided non-immunized controls in the assays, and the APR indices were referenced to species-typical hematology norms generated from 40 non-immunized healthy adult females. All staff wore masks and other personal protective equipment (PPE) to prevent exposure to new respiratory pathogens, such as SARS-CoV-2. No natural COVID infections have ever been detected in this monkey colony, in keeping with surveillance testing for new viral infections in other macaque colonies [37,38]. During this experiment, each monkey was housed socially as a pair with one other monkey. All research procedures were approved by the Institutional Animal Care and Use Committee (L006578).

### 2.2. Blood Collection

Small blood samples (<4 mL) were collected in serum-separator vacutainers (Becton Dickinson, Franklin Lakes, NJ, USA) to establish a pre-immunization baseline and then at 2-week intervals for 3 months to assess primary and secondary antibody responses (see Figure S1 for a visual illustration of the sampling schedule). Serum aliquots were stored frozen at −70 °C and used to determine antibody inhibition of ACE2-RBD binding with RBD from the ancestral Wuhan strain and 5 other viral variants. One additional blood sample was collected 4–6 months after the initial immunizations to evaluate the persistence of T-cell memory responses. To evaluate inflammatory aspects of the systemic response to the vaccine and assess the APR after the primary and secondary immunizations, small blood samples (2 mL) were also collected on the morning after and at one week and divided into two 1.0 mL aliquots for blood chemistry and complete blood counts. All samples were obtained using a special apparatus designed for safe immobilization to avoid the use of sedating drugs that could potentially affect the monkeys’ immune responses during this experiment.

### 2.3. Immunization

The 12 monkeys were administered intramuscular injections of a sterile suspension of adjuvanted recombinant receptor binding domain–human IgG1-Fc fusion protein (RBD-Fc) containing ancestral Wuhan protein sequences (aa331–537) (Figure S1B). Six monkeys received a 2% alum suspension in PBS; 6 were administered a 1:1 suspension of AS03 in PBS. Specifically, at each of the three RBD-Fc doses, two monkeys were immunized with either alum or AS03 suspensions. Details about the synthesis protocol for producing a discrete region of the spike S1 subunit, the extended C-terminal domain containing the RBD, have already been reported [39]. The 1 mL suspension contained either 12.5, 25, or 50 µg of RBD-Fc in phosphate-buffered saline (PBS), and four monkeys were vaccinated at each dose level. For injection, the suspension was divided into two 0.5 mL aliquots and administered bilaterally into each upper thigh. The response to the recombinant protein and activation of antigen-presenting cells were enhanced by including aluminum hydroxide (Alhydrogel 2% Al(OH)_3_, vac.alum.50, Invivogen, San Diego, CA, USA) or AS03 (vac.as03-10, Invivogen) in the PBS suspension. Alum is a semi-crystalline aluminum adjuvant that has been used historically in Tdap and pneumococcal vaccines; it has also been tested as an adjuvant in recombinant-protein vaccine studies in monkeys [40]. AS03 is an oil-in-water emulsion that has been used since the 1990s, including in some influenza and COVID recombinant protein vaccines; it has also been employed in vaccine studies in monkeys [41]. AS03 was developed originally by GlaxoSmithKline and is composed of squalene, α-tocopherol, and polysorbate 80 in a water emulsion [28,42].

### 2.4. Complete Blood Count and Blood Chemistry Panel

Serum iron levels and the NLR, two established APR indices of health and disease in monkeys [43], were determined before immunization and then on Days 1 and 7 after immunization (Figure S1C). Only small volumes (2 mL) were collected to not influence the immune responses after vaccination, and the fresh blood was delivered on the morning of collection to a CLIA-certified clinical laboratory for a Blood Chemistry panel and Complete Blood Count (CBC, UnityPoint Health-Meriter Lab, Madison, WI, USA). Blood chemistry measures were generated with an automated clinical chemistry instrument (Roche Cobas Pro; ISE, c503, and 3810 Analytical Units using Cobas Pro Integrated software). Serum iron values were employed as the primary bioindicator of the APR in our analyses. The CBC with differential was determined with an automated hematology analyzer (Sysmex XN-10). The extent of the leukocytosis and shift in the Neutrophil-to-Lymphocyte Ratio (NLR) provided confirmatory evidence of a generalized acute phase reaction on the day after immunization.

### 2.5. Cutaneous Assessment Post Immunization

The vaccine injection site on each thigh was examined visually and manually when blood samples were collected on the mornings of Days 1 and 7 after the primary and booster immunizations. These examinations verified the absence of significant adverse effects that would be evident by erythema and induration, as well as by a heightened tactile sensitivity and/or leg withdrawal in response to manual palpation.

### 2.6. Antibody Assay

Prior to immunization, the monkeys were screened with the Colony Surveillance Assay SARS-CoV-2 kit (CSA:SARS-CoV-2, Intuitive Biosciences, Madison, WI, USA) to ensure all 12 were seronegative and had not been exposed previously to SARS-CoV-2. The CSA:SARS-CoV-2 is a sensitive test for identifying antibodies to SARS-CoV-2 spike S1 and S2 subunits and nucleocapsid antigens [39]. It is designed in a 96-well, multiplex format for simultaneous detection of antigen-specific antibodies in sera. The image capture system employs a CCD camera to quantify the signal, which is calibrated in Relative Intensity Units (RIUs). Specifically, the signal and calculated RIU are higher when there is more bound antibody. The assay uses SARS-CoV-2 recombinant proteins (S1, aa16-685; S2, aa686-1213; and nucleocapsid, aa1-419) expressed from CHO or HEK cells to ensure proper glycosylation. Because antibodies to spike S2 and nucleocapsid were not detected, our report focuses only on IgG responses to the S1 subunit. The initial screening of sera for confirmation of the monkeys’ seronegative status was conducted at the standard 1:100 dilution. Sera from the monkeys after immunization were tested at 3 dilutions: 1:1000, 1:10,000, and 1:100,000. We show antibody responses at the 1:10,000 dilution because it effectively reflected the trajectory of the antibody levels during both the primary and secondary responses, although high titers were also evident at the 1:100,000 dilution after the booster immunization.

### 2.7. ACE2-RBD Binding Inhibition

Biological activity of the antibody post immunization was evaluated by the amount of interference with the binding of recombinant ACE2 with SARS-CoV-2 spike protein (SP) and RBD antigens (COVID-19 Neutralization Assay, MesoScale Discovery, Rockville, MD, USA). Details about the specificity and sensitivity of this immuno-electrochemiluminescence platform [44,45], and the specific assay protocol [46], have been reported previously. In brief, sera were run at 1:100 and 1:1000 dilutions and tested against an antigen panel with 6 viral variants: Wuhan, Alpha, Beta, Gamma, Delta, and Omicron (MSD Panel #K15562U-2). Following the manufacturer’s instructions, the assay diluent was used as the Negative control. Percent inhibition (%) was calculated by comparing the binding inhibition (BI) elicited by sera with the response to diluent alone. If an interfering molecule was present, including RBD-specific IgG, the binding of RBD with ACE2 was inhibited. Additional negative assay controls included blood samples from non-immunized monkeys. Positive assay controls included anti-SARS-CoV-2 SP RBD neutralizing antibody (Human IgG1, ACRO Biosystems, SAD-S35, Lot # S35- 211VF1-VT) and a pooled human IgG-positive standard from patients who had been infected with SARS-CoV-2 (Frederick National Laboratory, Lot #: COVID-NS0109). The positive controls validated assay performance but were not needed to calculate the BI for the experimental samples.

### 2.8. ELISpot Assay of Cellular Memory

An additional 7 mL blood sample was collected by femoral venipuncture >4 months after the initial immunization to assess the persistence of T-cell memory. The general methods of the ELISpot protocol have been reported previously [47]. In brief, we used freshly isolated PBMC on the morning of collection from the EDTA-anticoagulated blood samples. The cells (1–2.5 × 10^5^ PBMC/well) were stimulated in vitro with 2 µg of SP from either the Wuhan or Gamma variant diluted in a DMSO-PBS solution (1:2), and the number of IFNg-positive cells per mL quantified in triplicate determinations after overnight incubation. IFNg-expressing cells were detected using ELISpot PLUS kits (MABTECH Inc, Cincinnati, OH, USA) according to the manufacturer’s recommendations. Wells were imaged and spot-forming cells (SFCs) counted with an AID ELISPOT reader (AID, Strassberg, Germany). Negative and positive controls were included in each assay. Values were considered positive when the mean number of SFCs from test wells was at least twice the mean plus two standard deviations of background values for the control wells without SP. The criterion for a positive response to SP was >50 spots/million cells. To identify the maximal cellular response for this assay, monkey PBMCs were also stimulated in another control well with 10 μg/mL Concanavalin A (Sigma-Aldrich, Burlington, MA, USA).

### 2.9. Statistical Analyses

Descriptive statistics, including means and variance, were examined before statistical testing to verify the absence of extreme outliers. In addition to the arithmetic means and variance estimates illustrated in the figures, geometric means and confidence intervals are provided for the primary variables in the Supplementary Materials (Tables S1 and S2). Antibody responses to primary and secondary immunization with RBD-Fc were evaluated with repeated measures analysis of variance (ANOVA) to assess temporal changes. The *p*-value for the repeated measures factor was adjusted with the Greenhouse–Geisser correction to lessen the chance of false-positive results. After a significant main effect was obtained, differences at specific time points were examined with *t*-tests, adjusting the *p*-value for pairwise testing by the possible number of planned comparisons (e.g., baseline vs. 5 post-immunization time points). Type of adjuvant and RBD-Fc concentration were considered as between-factors. Post hoc testing of significant between-factors was performed with the Scheffe test. Similar ANOVAs were used to assess the APR (i.e., changes in serum iron and NLR) on the morning after and one week after the primary and secondary immunizations. The analysis of antibody inhibition of ACE2-RBD binding included time post immunization (i.e., 4, 8, and 12 weeks) and the extent of inhibition across the 6 viral variants that were tested. Because of the large difference in the amount of blocking of ACE2-RBD binding by the two serum dilutions used in the assay (i.e., 1:100 and 1:1000), the BI values for the two dilutions were analyzed separately. Analysis of the ELISpot results focused first on verifying that there was activation of IFNg-positive cells and if there was a significant difference in PBMC activation after stimulation with Wuhan or Gamma SP. Then, the correlation between IFNg-positive cells activated by Wuhan SP and individual variation in the decrement in serum Fe after immunization was examined. Pairwise correlations were examined with Pearson’s r tests. When multiple correlations were conducted, the Bonferroni method of adjusting the *p*-value for attaining significance by the number of planned comparisons was applied (e.g., associations for ACE2-RBD BI testing of 6 variants). Alpha for significance was set at *p* < 0.05.

## 3. Results

### 3.1. Antibody Response to Immunization

**Figure 1:**
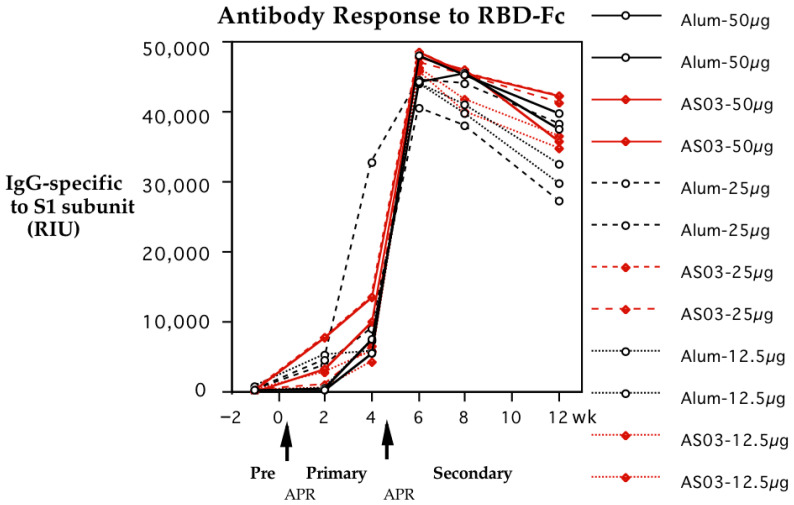
Primary and secondary antibody responses of rhesus monkeys after vaccination with recombinant spike protein–fusion protein (RBD-Fc) in alum or AS03 suspensions. All monkeys had been screened prior to immunization (Pre) to verify they had not been exposed previously to SARS-CoV-2. The timing of the primary and secondary immunizations is illustrated with arrows. The second vaccination was administered after the blood samples were collected at 4 weeks. Blood samples to assess the APR were collected on Day 1 and Day 7 after each vaccination. Responses are shown for 12 monkeys: 6 in each adjuvant condition and 4 at each of the 3 concentrations of RBD-Fc: 12.5, 25, and 50 µg/mL. Antibody titers to the SP subunit 1 were quantified in relative intensity units (RIUs) and are shown for the 1:10,000 serum dilution. Antibody levels increased over time, and the secondary response was significantly higher during the 2 months after the booster immunization (*p* < 0.001).

All 12 females were verified to be seronegative for SARS-CoV-2 S1 and S2 subunits and nucleocapsid prior to immunization. After administration of the adjuvanted recombinant RBD-Fc vaccine containing either alum or AS03, antibody to the RBD of the S1 subunit increased significantly across the 3-month assessment (F{5, 30} = 471.26, *p* < 0.001). Antibody was already detectable at 2 weeks after the first immunization, and the titers were significantly above the pre-immunization background levels (*p* < 0.03). As shown in Figure 1, large increments in antibody levels were elicited by the second immunization with RBD-Fc, peaking at 2 weeks after the booster. Because robust antibody responses were already elicited by immunization at the lowest RBD-Fc concentration of 12.5 µg, the antibody titers did not differ significantly across the three dose levels (i.e., 12.5, 25, and 50 µg/mL) (Figure S2). The type of adjuvant also did not significantly affect the antibody responses (Figure S2, Table S1).

### 3.2. Cutaneous Reaction to Immunization

Manual and visual inspection of the vaccine injection sites on the monkeys’ thighs did not reveal any erythema or swelling, nor overt signs of infection on Days 1 and 7 after immunization. In addition, none of the monkeys evinced a heightened tactile sensitivity or behavioral reactions suggestive of discomfort in response to gentle manual palpation of the injection sites after the primary or secondary immunizations.

### 3.3. Acute Phase Reaction (APR)

Nevertheless, the blood samples obtained on the following morning after immunization indicated that the APR metrics were markedly different from the species-typical values prior to immunizations (Table S2). At one week after immunization, both the serum iron level and the NLR had returned to the normal range and were no longer different from pre-immunization levels. The decreases in serum iron observed after vaccination did not differ significantly between the two adjuvant suspensions (Table S3). Statistically significant declines in serum iron occurred on Day 1 after both the primary and secondary immunizations (*p* < 0.0001) and did not differ significantly in magnitude. The changes in serum iron were associated with the increase in the total leukocyte count, as well as a pronounced neutrophilia and higher NLR on the morning after vaccination (Figure S3). The high NLRs on Day 1 were significantly different from pre-immunization values and the follow-up levels at one week, when they had returned to the species-typical range (*p* < 0.002). Administering the RBD-Fc in Alum or AS03 suspensions did not differentially affect the magnitude of the transient neutrophilia and lymphocytopenia on Day 1 (Table 1). A subgroup analysis comparing the APRs in response to the three different concentrations of RBD-Fc is provided in Table S3. Although there was a trend for a smaller decrement in serum iron and smaller shift in the NLR after administration of RBD-Fc at 12.5 µg/mL, post hoc testing indicated the differences were not significant when compared to the APRs elicited by the two higher RBD-Fc doses at 25 and 50 µg/mL.

### 3.4. Inhibition of ACE2-RBD Binding

**Figure 2:**
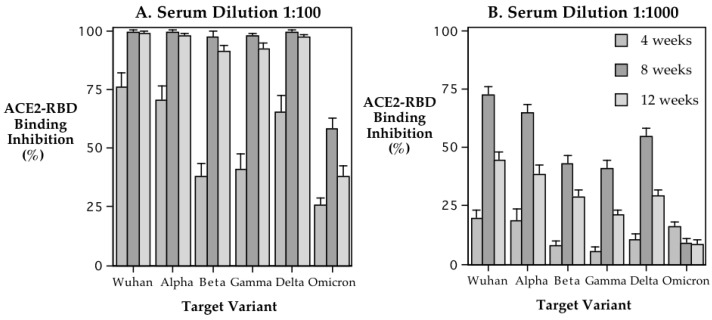
Inhibition of ACE2-RBD binding at 4, 8 and 12 weeks after the primary immunization. The 8- and 12-week time points were one and two months after the booster immunization, respectively. (**A**) Mean (+S.E.M.) BI values for 6 SARS-CoV-2 variants are shown at the 1:100 serum dilution recommended by the manufacturer of the assay kit. Strong inhibition of ACE2-RBD binding was prominently evident for Wuhan and other early variants, including the Delta variant, but reduced for Omicron. (**B**) Mean (+S.E.M.) BI when sera were diluted further to 1:1000. Maximal BI was found with sera at 8 weeks after the initial immunization. The BI values from this time point at the 1:1000 serum dilution were used for examining the extent of the association with individual variation in the APR on the day after immunization (Figure 3).

After immunization, the sera from the monkeys were highly effective at blocking the binding of RBD to the soluble ACE2 receptor when tested in the BI assay (Figure 2). The extent of this inhibition was significantly influenced by time after immunization. Maximal BI was seen at Week 8, which was one month after the booster immunization. More inhibition was exhibited by sera from monkeys vaccinated with the alum-adjuvanted suspensions at week 4 after the primary immunization (*p* < 0.0001), but this difference from monkeys immunized with AS03 suspensions was no longer significant after the vaccine booster. Similarly, at 4 weeks after the primary vaccination, there was a significant effect of immunizing dose, with the most inhibition across all six viral variants by sera from the monkeys administered recombinant RBD-Fc proteins at 50 µg/mL (*p* < 0.003). However, the effect of immunizing dose was also no longer evident after the booster. Finally, more BI was evident against RBD from Wuhan and other early variants than for the Omicron variant when using the manufacturer’s recommended 1:100 serum dilution (F{5,36} = 15.65, *p* < 0.0001). Because of the extremely high level of inhibition found at the 1:100 dilution, we also tested whether biological activity would continue to be evident at a dilution of 1:1000 (Figure 2B). Even with more diluted sera, we observed high levels of inhibition of ACE2-RBD binding for the early variants, especially at week 8 after the primary immunization (i.e., with sera collected at one month after the booster). The significant reduction in the inhibition of ACE2-RBD binding when tested with SP from the Omicron variant was particularly evident when the sera was more dilute and assayed at 1:1000.

**Figure 3:**
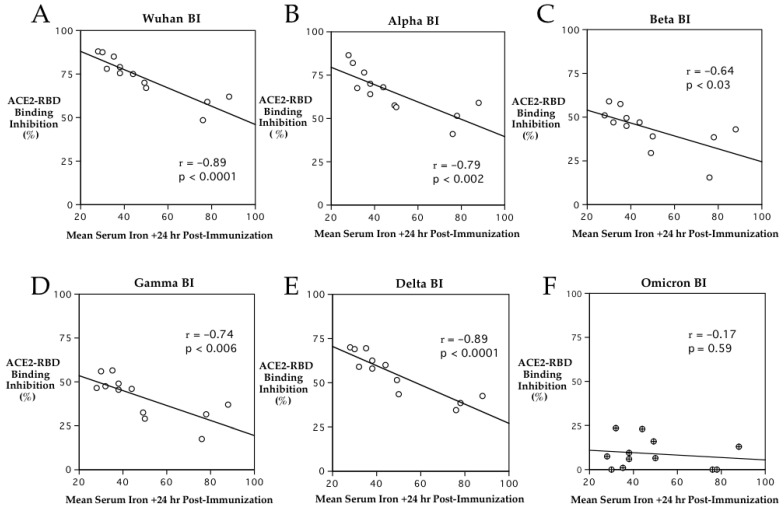
Correlation between mean serum iron level on the morning after immunizations (i.e., serum iron at 24 h after the primary and secondary vaccination/2) and the inhibition of RBD-Fc binding. Results are shown for SP RBD from the ancestral Wuhan strain and 5 other variants. All monkeys had been immunized with RBD-IgG1-Fc fusion protein containing ancestral Wuhan protein sequences. Binding inhibition (BI) values are from the testing of a 1:1000 dilution of sera at 8 weeks after the initial immunization (i.e., at one month after the second vaccination) (see Figure 2B for mean amount of BI at 2 other timepoints after immunization for all 6 viral variants). Lower serum iron, which reflected a larger APR, was associated with more binding inhibition against RBD from Wuhan and other early variants Figure 3A–E). However, the association was not evident with the low inhibition of ACE2-RBD binding for the Omicron variant (Figure 3F). The correlations between the decrement in serum Fe after vaccination and the inhibition of ACE2 binding with the RBD from Wuhan SP are shown at 2 time points after immunization in Figure S4.

Figure 3 shows the association between each monkey’s serum iron level on the mornings after the primary and secondary immunizations and the individual variation in inhibition of ACE2-RBD binding after the booster vaccination (i.e., week 8 after the initial immunization). As can be seen, monkeys with the lowest serum iron levels indicative of larger APRs after immunization also had serum antibody that exerted more inhibition of ACE2-RBD binding. The correlations for Wuhan and three other early viral variants including Delta were statistically significant (Figure 3A,B,D,E). The association with the Beta variant was similar, albeit with a lower r-value. After adjusting the *p*-values for multiple comparisons and the possibility of a false-positive, it no longer retained statistical significance (Figure 3C). The association with the serum iron level was not evident with the low inhibition of ACE2-RBD binding against the Omicron variant (Figure 3F).

### 3.5. Cell-Mediated Immunity

**Figure 4:**
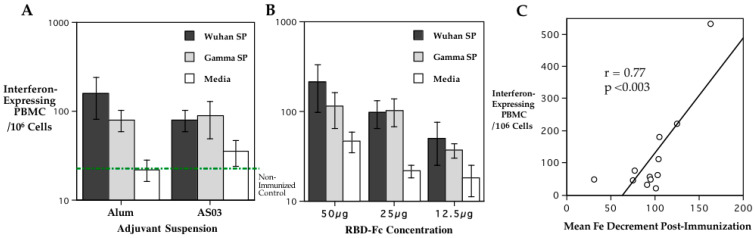
Cell-mediated memory was assessed by quantifying the number of IFNg-expressing PBMCs using ELISpot methods. (**A**) Significantly more IFNg-positive cells were present in wells stimulated with spike protein (SP) from the Wuhan and Gamma variants than in the control wells with just media (*p* < 0.01). PBMCs from a non-immunized monkey were also included as an additional assay control (green line). The cellular responses after stimulation with Wuhan and Gamma SP did not differ significantly between monkeys immunized with Alum and AS03 adjuvant suspensions. (**B**) Monkeys immunized with higher concentrations of RBD-Fc (25–50 µg/mL) evinced more antigen-reactive PBMCs after incubation with SP from the Wuhan and Gamma variants (*p* < 0.05). (**C**) Larger decrements in serum iron (Fe) after immunization (i.e., pre-immunization serum Fe minus serum Fe on the morning after immunization) were correlated with the number of IFNg-expressing cells after incubation with Wuhan SP. The scatterplot illustrates the mean number of activated cells as circles for all 12 immunized monkeys. The PBMCs for each monkey were assayed in triplicate.

Following the evaluation of antibody responses, one additional blood sample was collected >4 months after the initial vaccination to assess the persistence of cell-mediated immunity. PBMCs were stimulated in vitro with spike protein from the Wuhan or Gamma variants, and the number of IFNg-expressing cells quantified with an ELISpot protocol. All immunized monkeys were positive responders, and the number of IFNg-expressing cells provided evidence of sustained T-cell memory when compared to the cellular response in wells containing only control media (Figure 4A). The number of responsive cells was also higher when compared to the minimal cellular reaction of PBMCs from a non-immunized monkey in other wells on the microtiter plate. More IFNg-expressing cells were found after stimulation of PBMCs with Wuhan SP, especially for monkeys administered the higher concentration of RBD-Fc proteins in the alum suspension (Figure 4A,B). However, the more novel finding was when the number of IFNg-expressing cells was examined with respect to the variation in serum iron levels after immunization (Figure 4C). Across the 12 monkeys, the ones who had evinced larger decrements in serum iron also had more reactive PBMCs, resulting in a higher number of IFNg-expressing cells after stimulation with SP from the Wuhan variant (r = 0.77, *p* < 0.003).

## 4. Discussion

These results indicate that the adjuvanted recombinant fusion protein formulations were highly immunogenic in monkeys, in keeping with the findings from rodent models and people, which have demonstrated that several different types of adjuvanted vaccines can elicit robust humoral and cellular immune responses against SARS-CoV-2 [48,49,50]. Following the booster immunization, we found that antibody was detectable in sera even at an extremely low dilution of 1:100,000. The antibody also inhibited SARS-CoV-2 RBD binding to soluble ACE2, its primary receptor. This biological activity was so substantial that interference with ACE2-RBD binding was evident at serum dilutions 10-fold lower than the 1:100 dilution recommended by the manufacturer of the assay kit, suggesting it could limit viral entry and provide effective protection. The immunostimulatory actions of RBD-Fc fusion protein may be due to providing more potential epitopes, as well as because it may facilitate endogenous uptake via antigen-presenting cells that express the Fc receptor. There may also be indirect benefits because the fusion of the RBD to a Fc domain could enhance solubility and stability. It is also important to highlight that the responses to the vaccine did not differ significantly when the RBD-Fc protein was administered in an alum suspension or when AS03 was used as the adjuvant. The RBD-Fc in both adjuvant suspensions inhibited ACE2-RBD binding, as well as resulted in responsive IFNg-positive cells when the monkeys’ PBMCs were incubated with viral SP. Adjuvants have also been shown to enhance heterotypical immune responses [9,51,52], which may help to explain the inhibition of ACE2-RBD binding we found with other early viral variants, including Alpha, Beta, Gamma and Delta, despite their mutations. Overall, these results in monkeys concur with studies in murine and rabbit models that found potent immunostimulatory actions of extended RBD-Fc formulations and demonstrated protection using in vitro virus neutralization assays and by infection with virus [48,53]. However, it should be acknowledged that an earlier study in nonhuman primates that evaluated the effectiveness of five different adjuvants for boosting immune responses to SARS-CoV-2 RBD, which was presented on a two-component nanoparticle, indicated that the formulations with AS03 elicited stronger immune responses and the most crossover protection [30].

The other novel finding from the current study was that individual variation in the initial APR after immunization was associated with immune responses to the vaccine. The significant correlation between the decline in serum Fe and the extent of inhibition of RBD-ACE2 binding is in keeping with the widely held belief that there is a relationship between vaccine reactogenicity and immunogenicity [31,32,54]. Clinical follow-ups of people who received mRNA COVID vaccines have indicated further that the linkage could go beyond laboratory test measures of the APR, and may include larger physical reactions and symptoms, such as malaise, fever, pain and erythema at the injection site [31,55]. We opted to use two sensitive bioindicators of the APR that are readily available to clinicians and veterinarians: the reorientation of iron metabolism and the pronounced neutrophilia that leads to a higher NLR. Iron-related indices, including the parallel rise in the intracellular ferritin, the storage form of iron, have long been known to be a key component of the APR, especially in response to bacterial infections and sepsis [56]. The decrease in serum iron on the morning after immunization was correlated with the extent of the elevation in the NLR and proved to be the more sensitive predictor of individual variation in humoral and cellular immune responses. Lower serum iron was associated with the extent of the inhibition of ACE2 binding with RBD of the Wuhan variant—the immunogen used in our vaccine. Further, lower serum iron was also positively associated with the ACE2-RBD binding inhibition against four early viral variants: Alpha, Beta, Gamma and Delta. The correlation was lost, however, when inhibition of ACE2 binding was tested with RBD from the Omicron variant. Biomarkers of iron regulation and anemia have also been employed as sensitive predictors of disease course and mortality in severe COVID patients [57]. Despite our ability to use serum iron and the NLR to calibrate how the APR influences immune responses to vaccination, it is important to reiterate that the mediators of an enhancement of antibody affinity would more likely be hepatic acute phase proteins like C-reactive protein and serum amyloid protein, which function as pattern recognition receptors and can directly augment innate immune responses [52,58].

Notwithstanding the uniqueness of our vaccine approach and the use of a primate model, it should be acknowledged that the sample size was extremely small, and thus, the results require replication. Our experiment was conducted primarily to establish the feasibility of using monkeys as a model to test different recombinant RBD-Fc formulations, but it also provided an opportunity to assess the association between vaccine reactogenicity and immunogenicity. We were able to demonstrate inhibition of ACE2 binding with RBD of several early viral variants after administering recombinant proteins from the ancestral Wuhan strain as the RBD-Fc immunogen. The expected reduction in the capacity of sera from immunized monkeys to block ACE2-RBD binding when tested against the Omicron variant was also found. This difference with the Omicron variant likely reflects the larger number of RBD mutations that have increased its transmissibility and capacity for antibody escape [59]. We should also acknowledge that the monkeys were all females. It is not known if there is a sex difference in inflammatory reactivity between female and male monkeys comparable to the differences between women and men, which seems to account for why women experience more post-vaccine symptoms acutely [60]. There is an extensive literature documenting sex differences in the immune responses of many animal species [61,62], and the levels of several proinflammatory cytokines do differ between female and male monkeys [63]. Monkeys can provide a valuable animal model for investigating this type of research question, because their APR is more like humans. For example, CRP is the prominent hepatic acute phase protein in monkeys and humans, whereas serum amyloid protein is the more responsive protein in mice [64]. Finally, it should be acknowledged that our evaluation did not include the gold standard of viral infection. We did not directly assess viremic control and the severity of symptoms. Monkeys are a limited and costly resource, constraining our capacity to include some relevant control conditions, such as examining the effects of administering adjuvant without immunogen. We also did not examine other APRs and measures of innate immunity, including complement activation, which have been found to be sensitive bioindicators of viral control and disease course in monkeys infected with simian immunodeficiency virus [65].

## 5. Conclusions

Immunization of rhesus monkeys with adjuvanted recombinant protein antigen comprised of receptor binding domain–human IgG1-Fc fusion proteins (RBD-Fc) containing protein sequences from the ancestral Wuhan variant elicited large and sustained antibody responses. In keeping with human clinical trials, the vaccine protocol was well tolerated and did not result in adverse effects despite eliciting a transient APR that was detectable on the morning after immunization [49,66,67,68]. Further, the observed inhibition of ACE2-RBD binding by vaccine-induced antibodies was indicative of a mechanism that could impede viral entry. Cell-mediated responses were still evident >4 months after immunization, indicative of the persistence of T-cell memory. Two biomarkers of the APR after vaccine administration—serum iron and the NLR—were predictive of the extent to which ACE2-RBD binding was inhibited and were associated with the number of IFNg-expressing PBMCs activated by incubation with viral spike protein. Other researchers have evaluated immunized monkeys and demonstrated that the enhancement of their immune responses by vaccines is sufficient to provide protection against a viral challenge with SARS-CoV-2 [69,70]. As a hopeful future direction, recombinant RBD-Fc proteins have also been shown to be effective when administered as nasal vaccines [71,72].

## Figures and Tables

**Table 1:** Mean (S.E.M.) serum iron (Fe), total leukocyte count, and neutrophil and lymphocyte percentiles prior to immunization and then on Days 1 and 7 after the primary and secondary vaccinations with RBD-Fc proteins. Changes in serum Fe and NLR on Day 1 were the two APR measures used to examine if individual variation in reactogenicity was associated with immune responses to the vaccine. Table S1 provides a similar summary for the 3 different RBD-Fc doses.

	Pre	Primary	Primary	Booster	Booster	
**APR Indices**	(Baseline)	Day 1	Day 7	Day 1	Day 7	*p*-value
**Serum Fe (µg/dL)**	145.6 (8.7)	47.3 (7.0)	144.6 (8.4)	50.1 (7.6)	139.6 (8.9)	<0.001
• Alum	142.7 (13.3)	46.7 (12.9)	132.3 (12.4)	47.5 (7.2)	134.3 (11.6)	
• AS03	148.5 (29.9)	48.0 (7.2)	156.8 (9.7)	52.7 (14.1)	144.8 (4.1)	
**WBC (×1000/µL)**	8.3 (0.6)	15.0 (1.5)	8.2 (0.6)	14.4 (1.1)	8.0 (0.5)	<0.053
• Alum	8.3 (0.7)	17.2 (2.3)	8.3 (0.8)	15.5 (1.6)	7.9 (0.6)	
• AS03	8.3 (0.9)	12.7 (1.5)	8.1(1.0)	12.8 (1.6)	8.1 (0.8)	
**Neutrophil (%)**	53.5 (2.9)	69.2 (3.3)	50.6 (3.2)	72.1 (2.7)	49.3 (3.1	<0.0001
• Alum	52.5 (4.5)	71.5 (6.1)	56.2 (4.6)	70.7 (3.2)	50.5 (3.5)	
• AS03	53.8 (4.1)	67.0 (2.8)	45.0 (3.4)	73.5 (4.5)	47.8 (5.5)	
**Lymphocyte (%)**	41.0 (2.9)	21.2 (2.5)	42.4 (2.9)	20.7 (2.8)	42.9 (3.0)	<0.0001
• Alum	40.8 (4.8)	18.5 (4.4)	37.0 (4.1)	21.0 (3.2)	40.3 (2.6)	
• AS03	41.2 (3.8)	24.0 (2.5)	47.8 (2.9)	20.3 (4.8)	45.5 (5.5)	
**NLR**	1.45 (0.2)	4.18 (0.72)	1.33 (019)	5.10 (1.30)	1.32 (0.24)	<0.0002
• Alum	1.46 (0.29)	5.20 (1.18)	1.68 (0.30)	4.04 (0.94)	1.32 (0.19)	
• AS03	1.45 (0.30)	3.16 (0.71)	0.98 (0.13)	6.16 (2.50)	1.33 (0.47)	

*p*-values are based on the significant effect of the Time factor in the repeated measures ANOVA after the Greenhouse–Geisser correction. Bold font for values on Day 1 after the primary and booster immunizations connotes significant differences from Pre and Day 7 that were verified by post hoc testing. The effect of adjuvant suspension (Alum vs. AS03; 6 monkeys in each adjuvant condition, respectively) was not statistically significant. The transient changes in the APR on the day after immunization did not differ between the two adjuvant suspensions.

## Data Availability

Requests for access to data should be directed to CLC.

## Supplementary Materials

The following supporting information can be downloaded at: https://www.mdpi.com/article/10.3390/vaccines14060523/s1. Figure S1: Research Plan. Figure S2: Mean antibody responses for experimental subgroups immunized with adjuvanted recombinant RBD-Fc proteins. Figure S3: Correlations between serum Fe and NLR on Day 1 after primary and booster immunizations. Figure S4: Association between serum Fe on the mornings after primary and secondary immunization and the extent of the inhibition of SARS-CoV2 RBD binding to ACE2. Table S1: Geometric mean titers (GMT) and 95% confidence intervals (CI) for monkeys administered adjuvanted recombinant RBD-Fc in either alum or AS03 suspensions. Table S2: Geometric means and confidence intervals for serum iron (Fe) and neutrophil-lymphocyte ratios used as APR indices in the analyses. Table S3: Serum Fe, total leukocyte count, and neutrophil and lymphocyte percentiles prior to immunization and after monkeys were administered 3 different concentrations of RBD-Fc proteins.
