# Supplementary material for: Larger Acute Phase Reactions Are Associated with Immunogenicity of an Adjuvanted Recombinant Receptor Binding Domain Protein Vaccine Against SARS-CoV-2 in Rhesus Monkeys
Source: Vaccines (Basel). 2026 Jun 11;14(6):523. doi: 10.3390/vaccines14060523 (PMC13308342; doi:10.3390/vaccines14060523)
Supplement: Supplementary file 1 [file vaccines-14-00523-s001.zip › vaccines-4303508-supplementary.pdf]

Coe, C.L.; Nimityongskul, F.; Lubach, G.R.; Luke, K.; Rancour, D.; Schomburg, F.M. Larger acute phase reactions are associated with immunogenicity of an adjuvanted recombinant receptor binding domain protein vaccine against SARS-CoV-2 in rhesus monkeys. *Vaccines* **2026**.

Research Plan

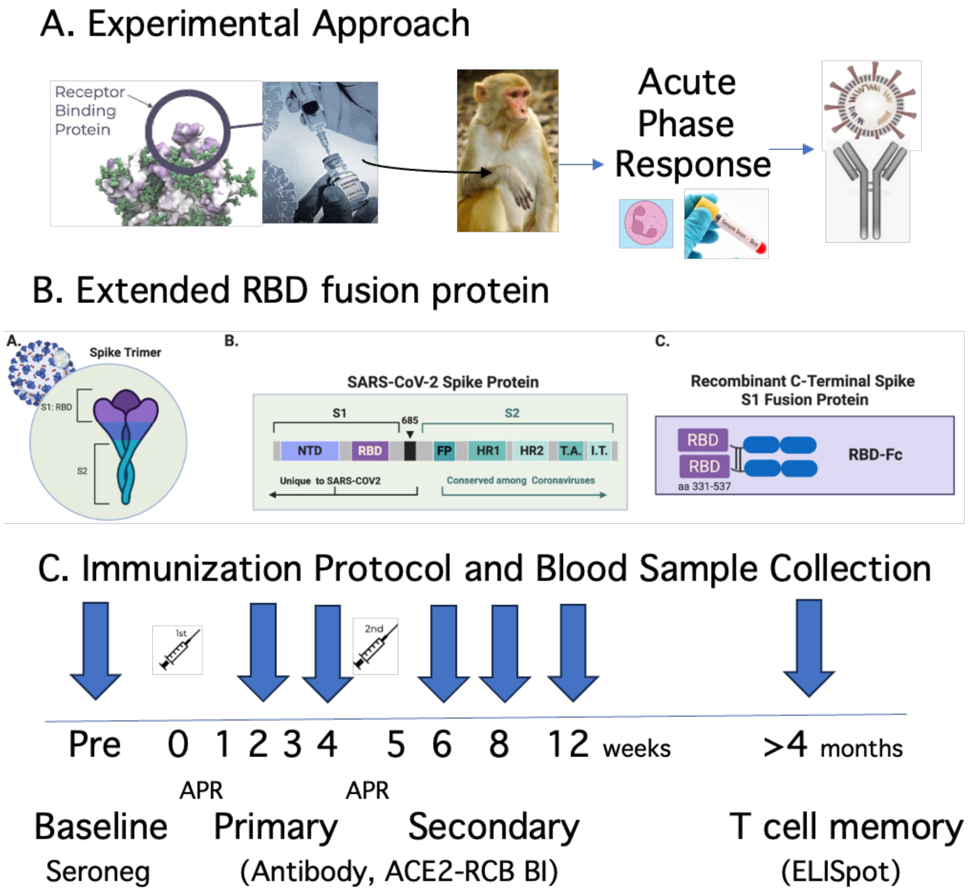

Figure S1. Illustration of the main research question, the recombinant fusion protein used for immunization, and blood sample collection schedule. **A.** The primary aim was to demonstrate that vaccination of rhesus monkeys with adjuvanted recombinant RBD-Fc proteins elicited robust RBD-specific IgG responses, and to determine the potential influence of the transient acute phase reaction (APR) on immune responses. **B**. SARS-CoV-2 structure and visual representation of the amino acids (aa331-537) in the RBD-Fc fusion protein. Image created in BioRender by Luke, K. (2026). **C.** Time points used to track the antibody response to the two immunizations. A baseline sample collected prior to vaccination verified that the monkeys were seronegative before RBD-Fc proteins were administered. None had antibody to the S1 subunit, S2 subunit and nucleotide antigens. Vaccine was administered twice, one month apart, as illustrated by the two syringe symbols. The APR was evaluated by determining serum iron levels and the neutrophil-to-lymphocyte ratio (NLR) on Days 1 and 7 during the week after the primary and secondary immunizations. The baseline samples prior to immunization (Pre) also provided negative control sera that were included with other negative and positive controls in the antibody and ACE2-RBD binding inhibition assays. The persistence of cell-mediated immunity was determined >4 months after immunization using an ELISpot protocol. Serum iron and NLR values prior to and after immunization were compared to normative hematological and blood chemistry values from 40 non-immunized, healthy adult female rhesus monkeys.

A.

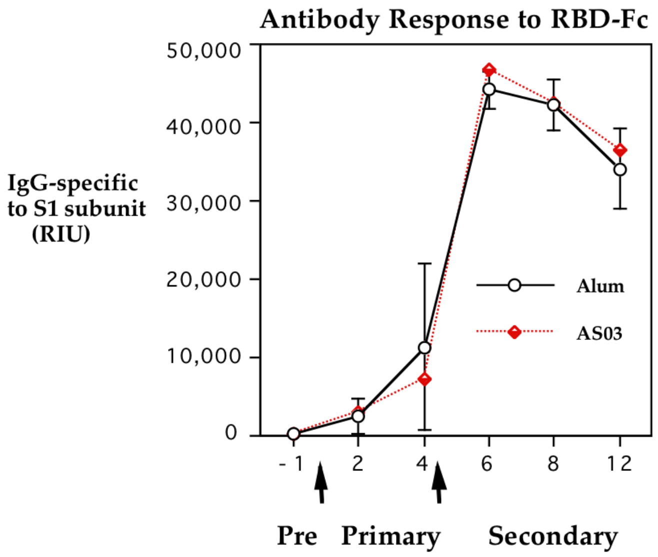

B.

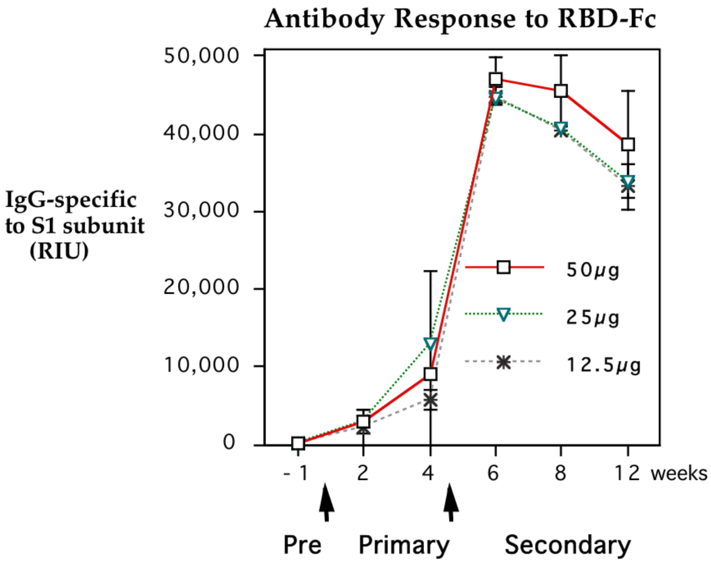

Figure S2. Antibody responses of monkeys immunized with adjuvanted recombinant RBD-Fc proteins. The arrows show when the primary and booster vaccines were administered. **A**. Mean (S.D.) responses for monkeys that were administered RBD-Fc proteins in either alum or AS03 suspensions (n = 6 and 6, respectively). B. Mean (S.D) responses of monkeys administered one of the 3 doses of RBD-Fc (n = 4 at each concentration). Antibody levels were quantified in Relative Intensity Units (RIU). There was a significant effect of time post-immunization, with higher titers after the booster vaccine, but the antibody responses did not differ significantly between the two types of adjuvant suspension. In addition, the monkeys' robust antibody response to the lowest concentration of RBD-Fc proteins (12.5 μg/mL) did not differ significantly from the overall antibody response at the highest concentration (50 μg/mL).

Table S1. Geometric mean titers (GMT) and 95% confidence intervals (CI) for monkeys administered adjuvanted recombinant RBD-Fc in either alum or AS03 suspensions. The values are expressed as Relative Intensity Units (RIU).

	Pre	2 wk	4 wk	6 wk	8 wk	12 wk*
		---Primary ---		--- Secondary ---		
Alum	27	1996	6555	46736	42172	36969
	(174)	(2054)	(12846)	(1893)	(1893)	(4081)
AS03	17	1247	7889	44020	42017	33658
	(53)	(1834)	(15462)	(837)	(3216)	(3871)

*Significant effect of Time post-immunization in the repeated measures ANOVA. Adjuvant formulation did not significantly affect the antibody response, nor was there a significant interaction between type of Adjuvant and Time. The statistical test results were similar when analyzed with log-transformed values. The antibody responses for all 12 immunized monkeys are illustrated in Figure 1 and portrayed for the two adjuvant conditions and 3 concentrations of RBD-Fc in Figure S2.

Table S2. Geometric means and confidence intervals for serum iron (Fe) and neutrophil-lymphocyte ratios used as APR indices in the analyses. Values are shown for the 12 monkeys in the vaccine study, before and after immunization, along with normative reference values for non-immunized adult female monkeys (n=40). Serum Fe and NLR values are shown for all monkeys in the vaccine study and then subdivided for the subset administered recombinant RBD-Fc proteins in either an alum suspension or AS03 suspension (n = 6 and 6, respectively).

	Reference Values	Pre-immunization	Post-immunization	
	Non-Immunized		Day 1*	Day 7**
Serum Fe	143 (7)	146 (17)	49 (11)	142 (14)
•Alum		136 (25)	47 (26)	137 (24)
• AS03		150 (19)	50 (21)	144 (24)
NLR	1.68 (0.25)	1.45 (0.39)	4.64 (1.72)	1.33 (0.32)
•Alum		1.39 (0.57)	3.15 (1.98)	1.37 (0.49)
•AS03		1.25 (0.57)	4.64 (1.72)	1.15 (0.59)

*Day 1 values after the primary immunization and secondary booster were averaged for comparison with reference values from non-immunized females.

** Day 7 values after the primary immunization and secondary booster were averaged for comparison with reference values from non-immunized females.

Serum Fe and NLR on Day 1 after vaccine administration was significantly different than the reference norms from 40 non-immunized female monkeys and from the baseline values of the 12 experimental monkeys prior to immunization. The APR indices on Day 1 values were also significantly different from the follow-up samples on Day 7 levels, which had returned to the normal range. The decrease in serum Fe and the increase in the NLR on Day 1 after immunization were not significantly different between monkeys administered the adjuvanted RBD-Fc vaccine in alum or AS03 suspensions.

Table S3. Serum iron (Fe), total leukocyte count, and neutrophil and lymphocyte percentiles prior to immunization and then on Days 1 and 7 after the primary and secondary vaccinations with RBD-Fc proteins for subgroups administered 3 different doses of RBD-Fc proteins. Changes in serum Fe and NLR on Day 1 were the two APR measures used to determine if individual variation in vaccine reactogenicity was associated with vaccine immunogenicity.

**Sample Time**	**Pre (Base)**	**Primary +1 day**	**Primary +1 week**	**Booster +1 day**	**Booster +1 week**	***P* value**
**Serum Fe (µg/dL)**	145.6 (8.7)	**47.3 (7.0)**	144.6 (8.4)	**50.1 (7.6)**	139.6 (8.9)	<0.001
• 50µg	159.3 (14.9)	42.5 (1.9)	157.3 (13.4)	35.0 (7.6)	163.8 (17.8)	
• 25µg	147.8 (11.9)	39.0 (9.8)	130.3 (17.8)	23.3 (13.5)	140.5 (9.6)	
• 12.5µg	1298 (17.7)	60.5 (18.8)	141.3 (12.1)	52.0 (17.8)	114.3 (6.8)	
**WBC (x10^3/µL)**	8.3 (0.6)	**15.0 (1.5)**	8.2 (0.6)	**14.4 (1.1)**	8.0 (0.5)	<0.053
• 50µg	8.2 (1.1)	16. 0 (2.3)	7.5 (1.2)	16.3 (2.4)	8.7 (1.1)	
• 25µg	9.1 (1.1)	17.2 (1.6)	9.5 (0.9)	14.7 (1.7)	7.7 (1.1)	
• 12.5µg	7.5 (0.8)	11.7 (6.5)	7.6 (0.9)	12.1 (1.9)	7.6 (0.4)	
**Neutrophil (%)**	53.5 (2.9)	**69.2 (3.3)**	50.6 (3.2)	**72.1 (2.7)**	49.3 (3.1	<0.0001
• 50µg	60.5 (5.0)	71.8 (4.0)	50.8 (6.8)	74.5 (3.1)	57.5 (6.9)	
• 25µg	50.3 (4.0)	73.3 (4.5)	51.0 (5.0)	69.5 (6.5)	46.3 (1.4)	
• 12.5µg	48.8 (5.1)	62.8 (7.8)	50.0 (6.4)	72,3 (4.8)	44.3 (5.0)	
**Lymphocyte (%)**	41.0 (2.9)	**21.2 (2.5)**	42.4 (2.9)	**20.7 (2.8)**	42.9 (3.0)	<0.0001
• 50µg	33.0 (6.1)	20.8 (4.4)	41.8 (5.4)	17. 8 (4.0)	35.3 (6.4)	
• 25µg	45.0 (3.7)	18.3 (4.2)	41.5 (3.2)	24.8 (6.8)	44.5 (0.9)	
• 12.5µg	45.0 (3.6)	24.8 (5.2)	44.0 (7.2)	19.5 (3.6)	49.0 (5.1)	
**NLR**	1.45 (0.2)	**4.18 (0.72)**	1.33 (0.19)	**5.10 (1.30)**	1.32 (0.24)	<0.0002
• 50µg	2.05 (0.40)	4.30 (1.36)	1.35 (0.34)	4.96 (1.20)	1.96 (0.62)	
• 25µg	1.16 (0.19)	4.98 (1.42)	1.28 (0.20)	5.98 (3.94)	1.04 (0.04)	
• 12.5µg	1.14 (0.23)	3.24 (1.18)	1.37 (0.48)	4.36 (1.21)	0.98 (0.24)	

*P values are based on a significant effect of the Time factor in the repeated measures ANOVA, using the Greenhouse-Geiser correction. The responses of all 12 monkeys were examined further by post hoc testing of the mean differences on Day 1 after each immunization, with the P values adjusted by the number of planned pairwise comparisons. Differences in the APR associated with the concentration of recombinant RBD-Fc were not statistically significant.

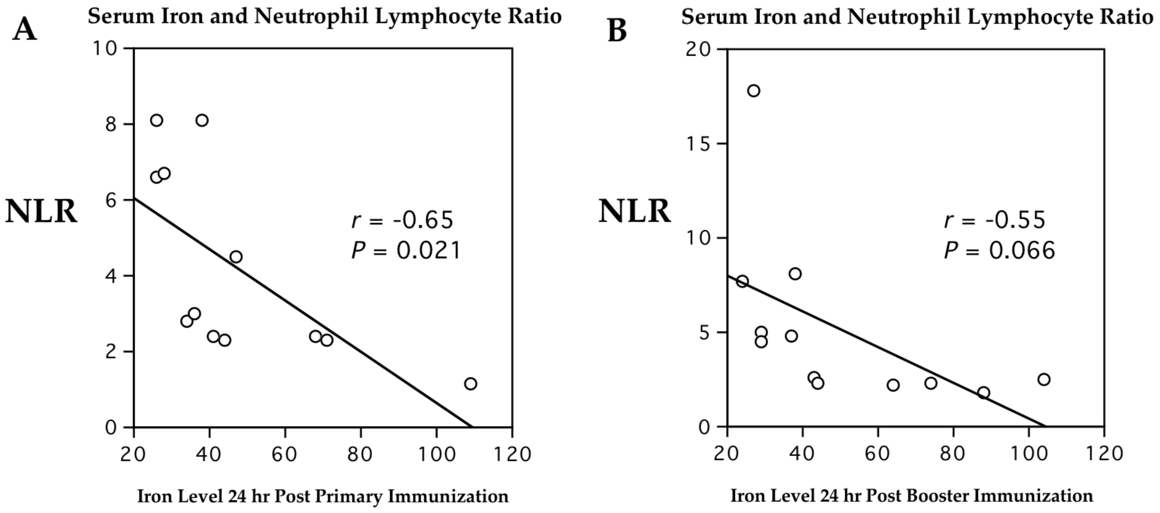

Figure S3. Two bioindicators of the acute phase reaction (APR) on the morning after the primary immunization (**A**) and after the booster immunization at Week 4 (**B**). The circles show the values for all 12 monkeys. Data are from the same 12 monkeys at two different time points in the immunization protocol (i.e., the day after the primary immunization and the day after the secondary immunization). The APR was of a similar magnitude after both immunizations. Serum iron levels on the morning following vaccination were significantly below the normal range for serum iron in rhesus monkeys (see **Table S2**). The monkeys with the lowest serum Fe also showed a larger increase in the NLR, although the moderate strength of the correlation suggests that the association likely reflects parallel changes and different regulatory pathways rather than a causal connection. In addition, the change in serum Fe appeared to be the more sensitive biomarker when assessing if individual variation in the APR was predictive of the monkeys' immune responses to vaccination. The decrease in serum Fe on Day 1 was associated with the extent of inhibition of ACE2-RBD binding as well as the activation of IFNγ-expressing PBMC by viral antigen in the ELISpot assay.

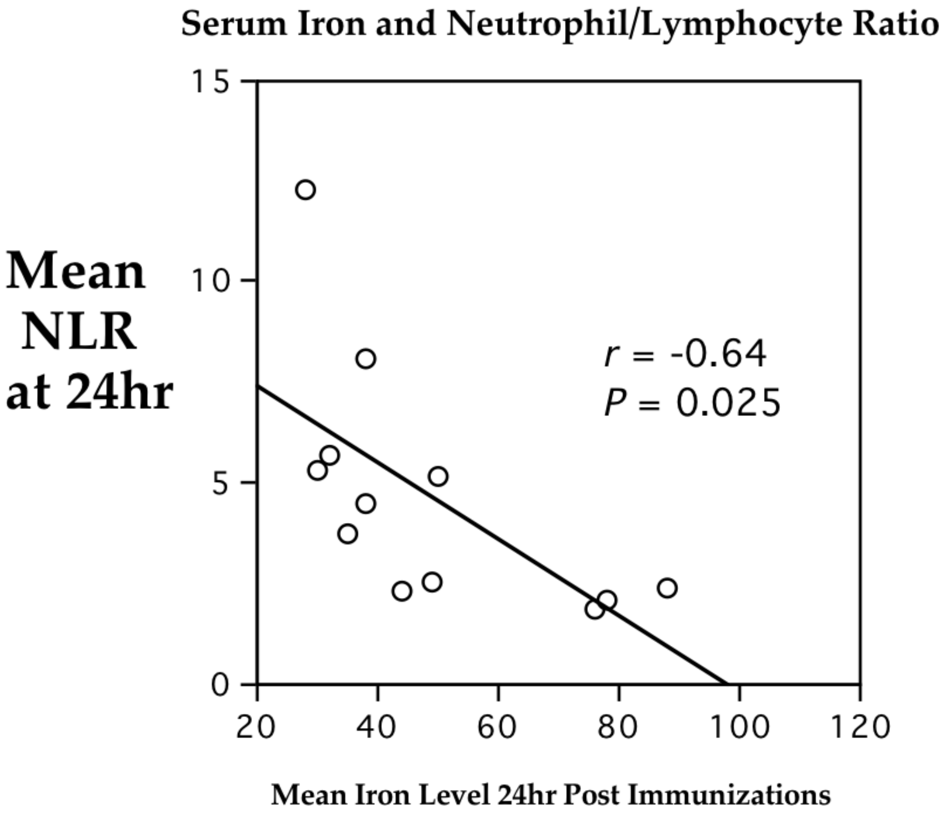

Figure S3C. Mean serum iron and the mean neutrophil lymphocyte ratio (NLR), averaged across the morning after the primary immunization and the morning after the secondary booster. The change in the APRs was similar after both immunizations. Each circle represents one immunized monkey and is the average of the values shown in Figures S3A and S3B. . Lower serum Fe levels were associated with a higher NLR. In addition, higher NLRs were associated with more inhibition of ACE2-RBD binding when tested with Wuhan SP ($r = 0.62$, $P < 0.031$). See **Figure S4** for the association between serum Fe levels post-immunization and the inhibition of ACE2 binding with the RBD from Wuhan SP.

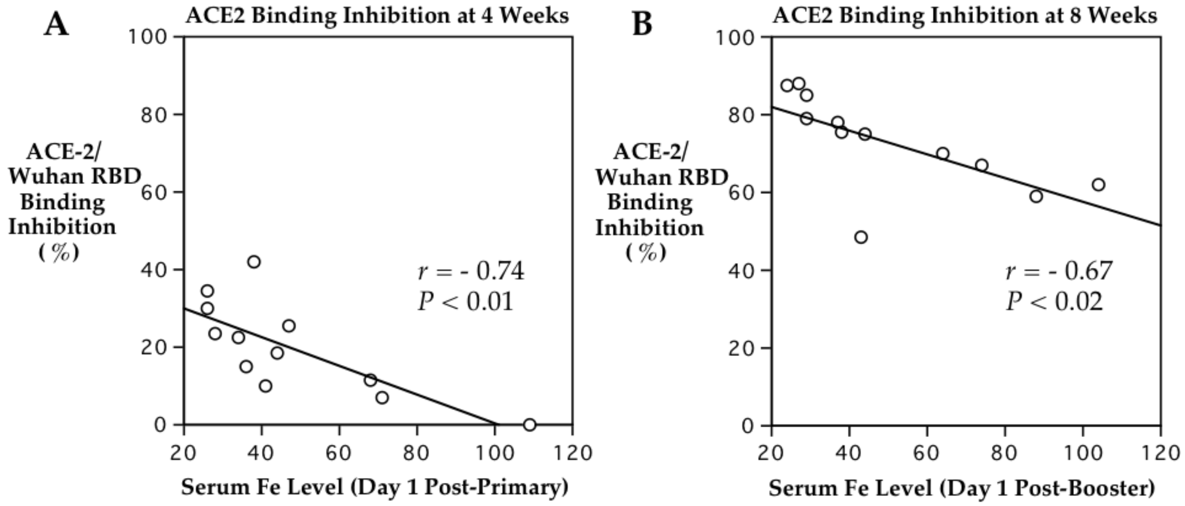

Figure S4. Association between serum iron on the morning after immunization and the extent of the inhibition of SARS-CoV2 RBD antigen binding to angiotensin-converting enzyme 2 (ACE2). **A.** The first panel shows serum Fe on the morning after the primary immunization and its association with the binding inhibition (BI) elicited by sera from the 4-week time point, one month after the first vaccine was administered. **B.** The second panel shows serum Fe levels on the morning after the booster vaccine and the BI elicited by sera obtained 4 weeks later during the secondary response (i.e., week 8 after the initial immunization). Sera were diluted 1:1000, which was 10-fold more than the manufacturer's recommendation. We diluted the monkeys' sera further because of their robust immune responses to the vaccine. **Figure S4** show the results only for the Wuhan variant, but the BI results for all 6 variants are shown in **Figure 2**. Significant inhibition was seen for early variants, including the ancestral Wuhan strain, Alpha, Beta, Gamma and Delta, but cross protection against Omicron was reduced. As a Negative control, the baseline sera from the 12 monkeys prior to immunization were also tested and elicited negligible BI (<1%). Minimal BI was also found with 3 other non-immunized serum samples, which provided additional control specimens. Two Positive controls were also included in the ACE2-RBD BI assay. Sera from our vaccinated monkeys exhibited more BI than both anti-SARS-CoV-2 spike RBD neutralizing antibody and a pooled human SARS CoV-2 national IgG positive standard at the dilutions tested. More inhibition of ACE2-RBD binding when tested with Wuhan SP was also associated with higher NLR values on Day 1 after immunization ($r = 0.62$, $P < 0.031$). This effect was in keeping with the inverse correlation between higher NLRs and lower serum iron levels.
